# Robotic‐assisted laparoscopic ureteral reimplantation using the LUAA technique: VCUG‐confirmed outcomes, learning curve and CUSUM analysis

**DOI:** 10.1002/bco2.70271

**Published:** 2026-08-27

**Authors:** Daniel Fu, Priyank Yadav, Jorge Arias, David Kim, Mohan Saheb Gundeti

**Affiliations:** ^1^ University of Chicago Section of Urology Comer Children's Hospital Chicago Illinois USA

**Keywords:** CUSUM analysis, learning curve, LUAA technique, paediatric robotic urology, robotic ureteral reimplantation, vesicoureteral reflux

## Abstract

**Objective:**

To delineate the learning curve of robotic‐assisted laparoscopic ureteral reimplantation (RALUR) through the LUAA extravesical technique, we characterized efficacy and safety outcomes throughout surgical proficiency development using cumulative sum (CUSUM) analysis and perioperative trifecta outcomes. RALUR is the minimally invasive alternative to open reimplantation for paediatric vesicoureteral reflux (VUR); however, its learning curve remains poorly defined due to small series and inconsistent outcome reporting.

**Patients and methods:**

We performed a retrospective single‐surgeon study of 125 patients undergoing RALUR for primary VUR divided into pre‐LUAA (2008–2011), evolving LUAA (2011–2014) and standardized LUAA (2014–2025) phases. The primary outcome was radiographic VUR resolution on postoperative voiding cystourethrogram. Secondary outcomes included urinary retention and 90‐day Clavien–Dindo grade ≥3 (CDG3+) complications. CUSUM analysis of operative time defined competency, proficiency and mastery.

**Results:**

VUR resolution improved from 76% (pre‐LUAA) to 85.2% (evolving) and 94.4% (standardized) with an overall success rate of 87.2%. Urinary retention (4%) and CDG3+ complications (3.2%) remained low. CUSUM analysis demonstrated competency at case 28 and mastery at case 99 overall. Limitations include retrospective design, single‐surgeon experience and lack of comparison with open surgery.

**Conclusions:**

This largest single‐surgeon study with objective VCUG‐based outcomes demonstrates that the LUAA technique achieves high radiographic success with low complication rates. Surgical proficiency is safely attained within a modest case volume for unilateral as well as bilateral cases. These findings provide a quantitative framework to guide training and credentialing in RALUR.

## 1 INTRODUCTION

Vesicoureteral reflux (VUR) is a common paediatric urologic condition affecting 0.4%–1.8% of the general paediatric population and identified in up to 33% of the children presenting with febrile urinary tract infections (UTI).^1^, ^2^ Left untreated, recurrent pyelonephritis in the setting of VUR carries a risk of progressive renal scarring and long‐term nephropathy.^3^ While low‐grade VUR may resolve spontaneously or be managed with antibiotic prophylaxis, high‐grade or persistent VUR often requires surgical intervention. Endoscopic injection of bulking agents offers a minimally invasive option, though with comparatively lower durability, particularly in high‐grade reflux.^4^ Open ureteral reimplantation remains the historical gold standard with radiographic success rates >95%^5^ but is associated with significant pain, longer hospital stays, urinary retention and visible scarring.

Robotic‐assisted laparoscopic ureteral reimplantation (RALUR) was first described in the early 2000s and has gained popularity as a minimally invasive alternative, offering enhanced dexterity, 3D visualization and precision in the paediatric pelvis. Comparative studies and meta‐analyses report radiographic success rates of 87–93% for RALUR, comparable to open surgery, with benefits of shorter hospitalization and improved cosmesis.^6^, ^7^, ^8^ However, variability in outcomes persists, largely due to differences in technique and the learning curve associated with robotic surgery.^7^

Despite growing adoption, the learning curve for RALUR remains poorly characterized. Existing studies are limited by small sample sizes, multi‐surgeon designs, heterogenous surgical techniques, outcomes and complication reporting and, most importantly, inconsistent definitions of surgical success—with majority relying on clinical or ultrasound‐based endpoints rather than objective radiographic resolution on voiding cystourethrogram (VCUG).^9^, ^10^ Cumulative sum (CUSUM) analysis is a validated statistical method for delineating phases of surgical learning and has been applied to a limited number of RALUR series, though none have followed a single surgeon's technical development to mastery with rigorous objective outcome measurement.^9^, ^11^

Here, we present the largest single‐surgeon cohort of patients undergoing RALUR for primary VUR to date, spanning 17 years of practice. During this period, the LUAA technique—defined by optimized detrusor tunnel **L**ength, **U**‐stitch inclusion, **A**pical alignment suture and **A**dventitia inclusion in detrusorrhaphy—was developed and standardized. The objectives of this study were to evaluate trifecta outcomes (radiographic VUR resolution, urinary retention and 90‐day Clavien–Dindo grade ≥3 complications) across sequential phases of technique development and to characterize the learning curve through operative time CUSUM analysis.

## 2 PATIENTS AND METHODS

### 2.1 Study design and patient selection

We performed a retrospective review of consecutive patients who underwent unilateral or bilateral RALUR for primary VUR at a single academic centre between November 2008 and August 2025 by a single fellowship‐trained paediatric urologist (M.S.G.). Patients with ureterovesical junction obstruction or prior open reimplantation were excluded. Institutional review board approval was obtained.

### 2.2 Surgical technique

All procedures were performed using the extravesical RALUR technique with the da Vinci robotic platform (Intuitive Surgical, Sunnyvale, CA, USA). The operative approach evolved over the study period into the standardized LUAA technique. A detailed description of the LUAA technique has been previously published.^10^

To reflect the sequential phases of technical development, patients were stratified into three temporal cohorts:

- Cohort 1 (pre‐LUAA, 2008–2011)
- Cohort 2 (evolving LUAA, 2011–2014)
- Cohort 3 (standardized LUAA, 2014–2025)

### 2.3 Perioperative management

Patient demographics, perioperative data and postoperative outcomes were extracted from electronic medical records and reviewed independently. Prior to discharge, the urethral catheter was removed, and a postvoid bladder scan was performed to confirm bladder emptying and adequate voiding. Renal and bladder ultrasounds were obtained at 1 month, 6 months, and 1 year postoperatively. VCUG was performed at 4–6 months after surgery in all patients.

### 2.4 Outcome measures

The primary outcome was radiographic resolution of VUR on postoperative VCUG. Secondary outcomes comprised the trifecta of (1) radiographic VUR resolution, (2) postoperative urinary retention and (3) 90‐day Clavien–Dindo grade ≥3 (CDG3+) complications. Urinary retention was defined as the requirement for re‐catheterization following an initial catheter‐free voiding trial, either during the index hospitalization or within the follow‐up period. Complications below a CDG3+ were recorded and characterized separately.

### 2.5 CUSUM analysis

**FIGURE 1:**
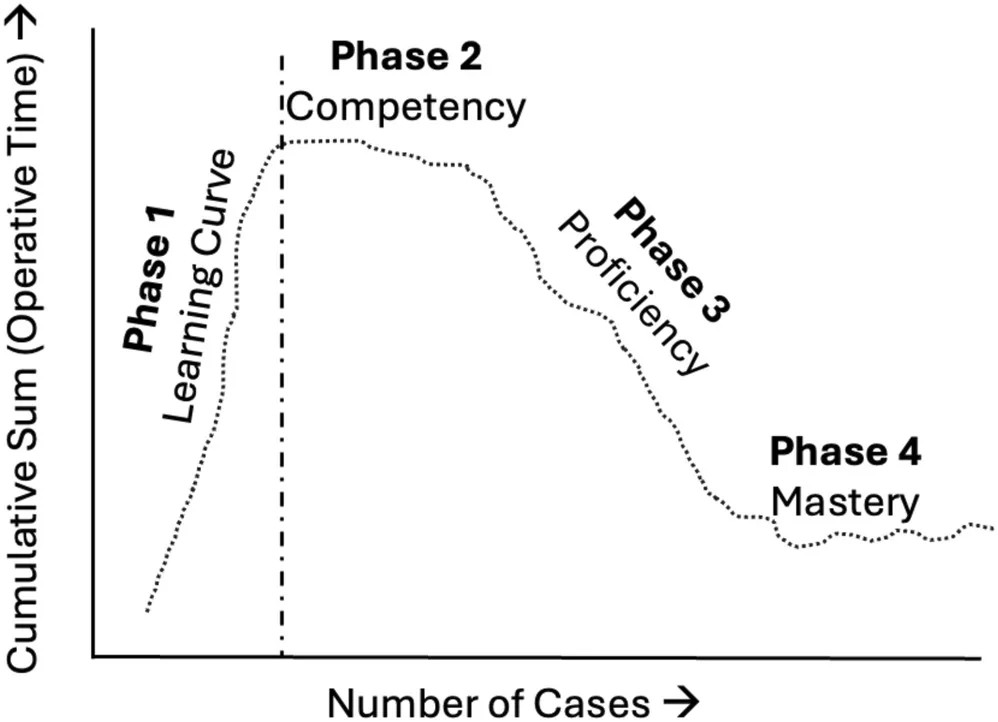
Representation of different phases of surgical skill acquisition in CUSUM analysis.

To delineate the learning curve, operative time was analysed using CUSUM methodology, a validated statistical technique for identifying systematic changes in surgical performance over time.^12^ Separate CUSUM analyses were performed for unilateral and bilateral procedures, ordered chronologically by date of surgery. For each case, the deviation from overall series mean operative time was calculated, and the CUSUM value was computed as the running sum of these deviations:

$$\text{CUSUMi}=\text{CUSUMi}-1+\left(\mathrm{OTi}-\mathrm{OT}®\right)$$

where OTi is the operative time of the *i*th case and OT̅ is the mean operative time across the entire series. The resulting CUSUM curves for unilateral and bilateral cases were superimposed chronologically for comparison. Four distinct phases were defined based on the morphology of the CUSUM curve (Figure 1):

- *Phase 1 (learning)*: Initial ascent to the peak, reflecting operative times exceeding the series mean.
- *Phase 2 (competency)*: Plateau near the mean, indicating achievement of procedural competency.
- *Phase 3 (proficiency)*: Downward slope below the mean, reflecting consistently shorter‐than‐average operative times.
- *Phase 4 (mastery)*: Final levelling and stabilization, reflecting consistent and reproducible performance.

### 2.6 Statistical analysis

Continuous variables are reported as mean ± standard deviation (SD) except for operative time, reported as median and interquartile ranges due to high variance. Categorical variables are reported as frequencies and percentages. Between‐cohort comparisons of continuous variables were performed using one‐way analysis of variance (ANOVA) and Kruskal–Wallis test, and categorical variables were compared using the chi‐squared test or Fisher's exact test as appropriate. A two‐sided *p*‐value of <0.05 was considered statistically significant. All statistical analyses were performed using DataTab (DataTab GmbH, Graz, Austria). CUSUM analysis was performed in Microsoft Excel.

## 3 RESULTS

### 3.1 Patient selection and baseline characteristics

A total of 141 patients underwent RALUR during the study period. Sixteen patients were excluded: 13 lacked a postoperative VCUG. Of the remaining three (all of whom converted to open surgery), one conversion was due to vascular injury during the pre‐LUAA period, one was due to ureteric injury, and one was due to leak at the detrusor hiatus (ureterovesical junction) requiring stent placement. The final cohort comprised 125 patients, of whom 41 (32.8%) underwent bilateral and 84 (67.2%) underwent unilateral ureteric reimplantation, representing a total of 166 ureteric units. Cohort 1 (pre‐LUAA, 2008–2011) included 25 patients, cohort 2 (evolving LUAA, 2011–2014) included 27 patients, and cohort 3 (standardized LUAA, 2014–2025) included 73 patients. The average follow‐up was 26 months, with a range of 0–156.7 months.

The mean age at surgery was 5.7 ± 3.2 years. The cohorts were well matched for age, sex, preoperative VUR grade and prior endoscopic injection (*p* > 0.05). The predominant preoperative VUR grades were grade 3 (41.6%) and grade 4 (43.2%), with grade 5 reflux present in 12.8% of ureteric units. Full baseline and perioperative characteristics are presented in Table 1.

**TABLE 1:** Preoperative and perioperative characteristics.

	2008–2011	2011–2014	2014–present	Overall	*p*‐values
No. of patients	25	27	73	125	
Total ureters	39	36	91	166	
Age (years), mean (SD)	5.5 (1.5)	5.7 (2.5)	5.8 (3.8)	5.7 (3.2)	0.921
Sex					
Male	4	8	19	31	0.488
Female	21	19	54	94	
Duplex ureter	7	5	17	29	0.720
Prior deflux injection	1	2	1	4	0.304
Redo reimplantation	0	0	3	3	0.335
Ectopic ureter	1	2	6	9	0.779
Preoperative VUR grade, number of ureters					
2	0	1	2	3 (2.4%)	0.600
3	8	13	31	52 (41.6%)	
4	15	9	30	54 (43.2%)	
5	2	4	10	16 (12.8%)	
Hydronephrosis SFU grade, number of ureters					
0	15	16	31	62	0.525
1	4	6	12	22	
2	3	2	18	23	
3	3	3	11	17	
4	0	0	1	1	
Operative time (min), median IQR	196 (181.5–227.5)	130 (105–167)	124 (107.5–144)	132 (110.5–175.5)	<0.001
Average follow‐up (SD), (months)	36.6 (41.1)	40.3 (43.7)	17 (17.5)	26 (31.9)	

Operative time decreased significantly across cohorts, from a median of 196 (181.5–227.5) min in cohort 1 to 130 (105–167) min in cohort 2 and 124 (107.5–144) min in cohort 3 (overall median 132 [110.5–175.5] min; *p* < 0.001).

### 3.2 Primary outcome: Radiographic VUR resolution

On a patient‐wise basis, radiographic VUR resolution on postoperative VCUG was achieved in 19 of 25 patients (76.0%) in cohort 1, 23 of 27 (85.2%) in cohort 2 and 67 of 73 (94.4%) in cohort 3, for an overall resolution rate of 109 of 125 patients (87.2%; *p* = 0.084). On a ureter‐wise basis, resolution was observed in 32 of 39 (82.0%), 31 of 36 (86.1%) and 80 of 91 (90.9%) ureteric units across the three cohorts, respectively, for an overall rate of 143 of 166 units (87.7%; *p* = 0.353). Trifecta outcomes across all cohorts are summarized in Table 2.

**TABLE 2:** Postoperative trifecta outcomes.

	2008–2011	2011–2014	2014–present	Overall	*p*‐values
Radiographic VUR resolution seen on VCUG (patient‐wise)	19 (76%)	23 (85.2%)	67 (94.4%)	109 (87.2%)	0.084
Radiographic VUR resolution seen on VCUG (ureter‐wise)	32 (82%)	31 (86.1%)	80 (90.9%)	143 (87.7%)	0.353
Post‐operative urinary retention	0	0	3 (4.1%)	3 (2.4%)	0.27
90‐day CDG3+	0	0	4 (5.5%)	4 (3.2%)	0.229

### 3.3 Secondary outcomes: Urinary retention and complications

Postoperative urinary retention occurred in one of 25 patients (4.0%) in cohort 1, none in cohort 2 and four of 73 (5.5%) in cohort 3, for an overall retention rate of five of 125 patients. Patient preoperative complexity is further described in Table 3, with four of five patients undergoing a bilateral operation, an age range of 2.9–5.8 years, an operative time range of 119–196 min and an average preoperative VUR grade of 3–3.5, and three patients with prior bowel bladder dysfunction. Notably, two of these cases of urinary retention resolved spontaneously without necessitating re‐catheterization or intervention, while the remaining three required placement of a suprapubic catheter or urinary catheter implantation. Urinary retention defined as requirement for re‐catheterization following a catheter‐free voiding trial was overall three of 125 patients (2.4%) and three of 73 in Cohort 3 (4.1%) for a *p* = 0.27. These patients are voiding via urethra, and none are on clean intermittent catheterization. Postoperatively, primary VUR was resolved for all patients who experienced urinary retention.

**TABLE 3:** Urinary retention preoperative complexity and postoperative results.

Case	Cohort	Unilateral vs. bilateral	Age (yr)	Duration of surgery (min)	Average VUR grade	Bowel bladder dysfunction	Toilet trained	Required catheterization	Time on catheterization	VUR resolution on VCUG
1	1	Bilateral	4.7	196	3	No	Yes	No	—	Yes
2	3	Bilateral	5.8	132	3	Yes	Yes	Suprapubic catheter placed	46 days	Yes
3	3	Bilateral	3.6	154	3.5	Yes	Yes	Urinary catheter placed	1 day	Yes
4	3	Bilateral	2.9	131	3	Yes	No	Suprapubic catheter placed	60 days	Yes
5	3	Unilateral	3.3	119	3	No	Yes	No	—	Yes

The overall 90‐day CDG3+ complication rate was 3.2%, all seen in cohort 3 (5.5%; *p* = 0.229). These comprised two cases of urine leakage, one case of UTI requiring hospital admission and one case of severe flank pain requiring hospital admission. Fifteen Clavien–Dindo complications below grade three were recorded in 14 patients (11.2%), consisting of 13 UTI (10.4%), one surgical site infection (0.8%) and one episode of cystitis (0.8%). Of 13 patients with UTI, 11 cases were within 90 days, all 13 were within 1 year. Seven were considered febrile UTIs (5.6%) while 6 were afebrile (4.8%). Two of these cases were recurrent, while the rest were isolated single cases (1.6%); recurrent UTI cases did not require interventions beyond antibiotics.

### 3.4 CUSUM analysis of operative time

**FIGURE 2:**
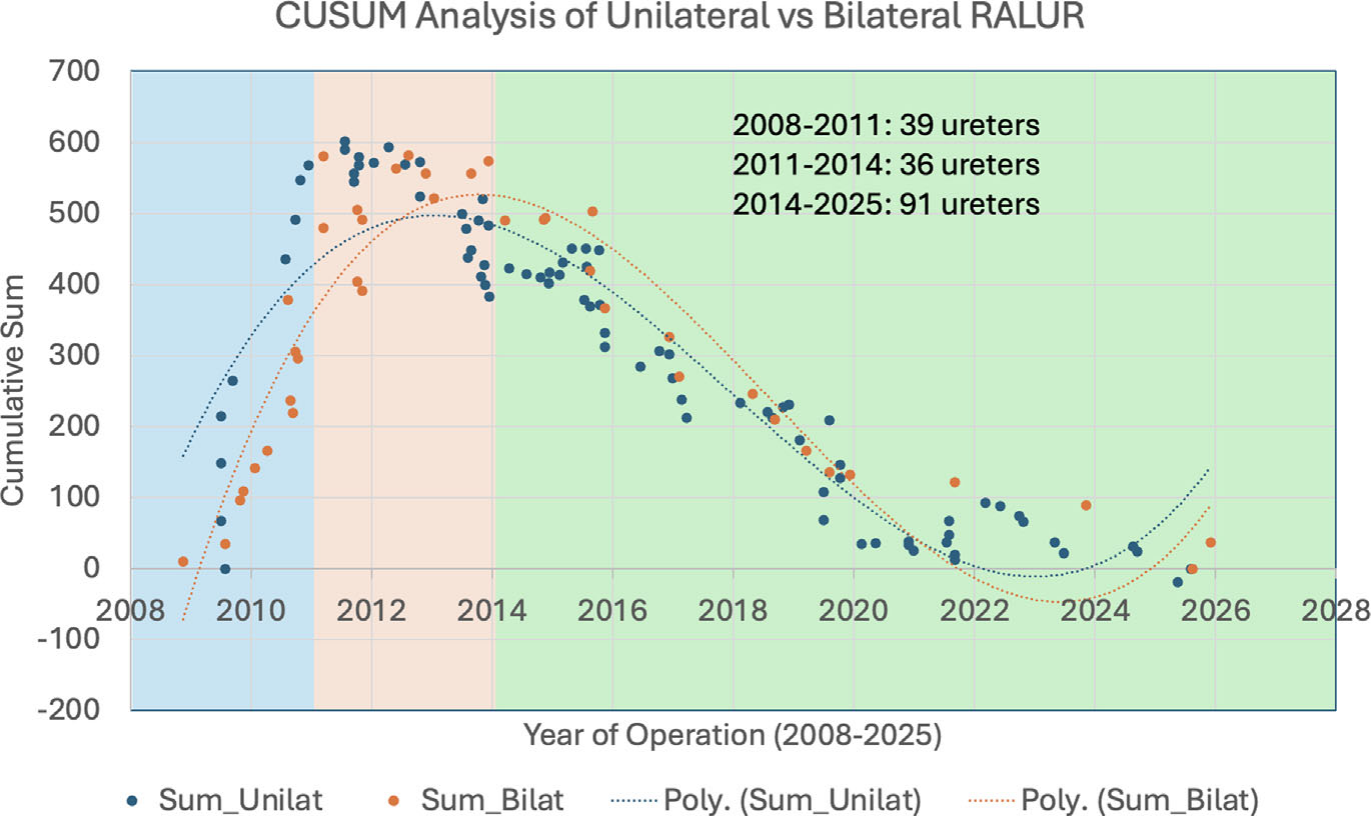
Unilateral and bilateral operative time CUSUM analysis.

CUSUM analysis was performed separately for unilateral and bilateral cases and superimposed chronologically. The resulting curve identified four distinct phases of learning for both unilateral and bilateral procedures (Figure 2).

The peak of the CUSUM curve—representing completion of the initial learning phase and transition to competency—was reached at case 28 overall (11 unilateral and 17 bilateral cases). The subsequent downslope reflected a phase of growing proficiency with operative times consistently falling below the series mean. Final plateau and stabilization of the CUSUM curve, indicative of mastery, was reached at approximately case 99 overall (65 unilateral and 34 bilateral cases). Notably, the bilateral CUSUM curve was shifted rightward relative to the unilateral curve; however, landmarks for competency and mastery occurred concurrently by operative date, indicating that technical development progressed in parallel for both procedure types.

## 4 DISCUSSION

This study presents the largest single‐surgeon cohort of RALUR with objective postoperative VCUG‐based outcome measurement, spanning 17 years of technical development. Using a trifecta framework of radiographic VUR resolution, urinary retention and 90‐day CDG3+ complications, we demonstrate progressive improvement in surgical outcomes across sequential phases of technique development, alongside a well‐defined learning curve characterized by CUSUM analysis.

### 4.1 Radiographic VUR resolution

The radiographic success rate in the standardized LUAA cohort was 94.4%, a meaningful improvement over the pre‐LUAA (76.0%) and evolving LUAA (85.2%) phases and compares favourably with published RALUR outcomes. Distinguishing radiographic from clinical success is essential, as definitions vary widely in the literature, complicating comparisons. Routine postoperative VCUG—the most objective and stringent measure—strengthens the validity of our results. Boysen et al. reported 87.9% radiographic resolution in 280 ureteric units,^6^ Esposito et al. noted 92% clinical success in 1362 patients (most studies lacking VCUG),^7^ and Gazzaneo et al. found 94.1% success for RALUR‐EV versus 92.5% for open reimplantation, with six of seven studies defining success radiologically.^8^ Collectively, these data confirm that the LUAA technique, once established, delivers radiographic success rates comparable to the best reported outcomes in the literature and equivalent to the historical gold standard of open reimplantation.

### 4.2 Urinary retention

Postoperative urinary retention is a well‐recognized complication of extravesical RALUR, attributed to inadvertent injury to the perivesical autonomic nerve plexus during dissection near the ureterovesical junction.^13^ Published retention rates following RALUR‐EV range from 0% to 40%.^6^, ^7^ In our series, the overall retention rate was 2.4%, with a rate of 4.1% in the standardized LUAA cohort. Notably, two of these cases of urinary retention did not require re‐catheterization and were self‐resolving. These cases were not associated with bowel bladder dysfunction, whereas the cases having a history of bowel bladder dysfunction and undergoing bilateral RALUR led to urinary retention requiring catheter placement. These are known risk predictors of urinary retention following RALUR, correlating with previous studies on this topic.^6^, ^14^ Although there was an absence of decreased urinary retention throughout the learning curve, this can be attributed to low sample sizes in the earlier cohorts or transient episodes of urinary retention without clinical significance. Overall, the rates of urinary retention are consistently low throughout the learning curve of the LUAA technique. We attribute these consistently low urinary retention rates to deliberate limitation of dissection near the ureteric insertion and avoidance of medial dissection, both of which are incorporated as standard elements of the LUAA approach.

### 4.3 CDG3+ complications and UTIs

The overall 90‐day CDG3+ complication rate of 3.2% in our series is comparable to published rates for both RALUR and open reimplantation. Suer et al. reported a CDG3+ rate of 5.2% following open ureteral reimplantation in 383 paediatric patients,^15^ while Boysen et al. reported a complication rate of 9.6% across all Clavien–Dindo grades in their multi‐institutional RALUR series (2.7% were CDG3+).^6^ Notably, all CDG3+ events in our cohort occurred in the standardized LUAA phase, which encompasses the largest number of patients; the absolute rate of 5.5% within this cohort remains within the range reported for open surgery. The absence of CDG3+ complications in the pre‐LUAA and evolving LUAA cohorts likely reflects both smaller sample sizes and different case selection rather than a true difference in complication risk. Importantly, the occurrence of CDG3+ complications did not increase during the earlier phases of the learning curve, confirming that the acquisition of RALUR proficiency was not associated with a period of elevated patient risk.

Additionally, clinical indications of success following RALUR further include the absence of recurrent UTIs. There are limited current data in the literature on rates of recurrent or febrile UTI following RALUR. Utilizing recurrent UTI as an indication for ureteral reimplantation, it is encouraging that the two cases in this learning curve were able to be managed conservatively without requiring surgical intervention. These low rates justify safety through the learning curve and identify complications to look out for that can be managed conservatively.

### 4.4 Learning curve and CUSUM analysis

Several systematic reviews and multi‐institutional studies have acknowledged the RALUR learning curve without specific quantification. Esposito et al. noted that learning curves are expected with new surgical procedures,^7^ while Gerber and Koh proposed that inter‐centre heterogeneity in RALUR outcomes may be partially attributable to the learning curve associated with robotic technology adoption.^16^ Our CUSUM analysis provides the most comprehensive characterization of the RALUR learning curve to date for a single surgeon, delineating four distinct phases from initial learning to mastery. Competency—defined as the point at which operative times plateau near the series mean—was reached at case 28 (11 unilateral and 17 bilateral cases). Mastery—defined as final stabilization of operative times at a consistently below‐average level—was achieved at approximately case 99 (65 unilateral and 34 bilateral cases). The concurrent development of competency and mastery in both unilateral and bilateral procedures, reflected in the parallel trajectories of the superimposed CUSUM curves, indicates that technical learning progresses simultaneously across both procedure types rather than sequentially.

Two prior studies reported CUSUM‐based learning curves for RALUR and merit direct comparison. Kim et al. achieved competency at case 23 in a series of 65 cases (27 unilateral, 38 bilateral) but showed no true downslope due to technique modifications and increased trainee involvement, precluding a reliable estimate of mastery.^9^ Importantly, this series did not incorporate routine postoperative VCUG, limiting the objectivity of reported success (Table 4). Shengkun et al. reported competency at case 11 for non‐tapered and case 9 for tapered reimplantation, with a proposed mastery at case 33 in a unilateral‐only series of 53 cases.^11^ We argue that their mastery point falls within our proficiency phase, occurring on the downslope of the CUSUM curve rather than at the final plateau. Furthermore, excluding bilateral cases underestimates the procedural complexity seen in clinical practice. Our series addresses both limitations by analyzing unilateral and bilateral procedures concurrently and applying objective VCUG‐based outcome measurement. Transient increases in operative time during the proficiency phase corresponded to platform transitions from the da Vinci Si to the da Vinci Xi system, reflecting real‐world adaptations to this long‐term single‐surgeon series.

**TABLE 4:** Surgical outcomes and learning curve data in studies addressing RALUR learning curve.

Study	Number of patients	Mean operating time (min)	Success rate	VCUG definition of success	Complication rate (CDG3+)	Follow‐up (mean/median)
Kim et al., 2025^9^	27 unilateral, 38 bilateral	157	93.8% (no re‐intervention); 83% (no UTI)	Not reported	6.2%	16.8 months
Shengkun et al., 2026^11^	53 unilateral	135	96.2% (radiographic); 90.6% (combined)	No grade 3–5 VUR	—	60 months
Present study Fu/Gundeti et al.	84 unilateral, 41 bilateral	145	94.4% for mature LUAA (radiographic)	No VUR	3.2%	26 months

The trajectory of our learning curve is likely multifactorial, with robotic surgical technical expertise and technique modification as potential contributions to developing excellence in this surgery. Notably, this reflects pragmatic practice within urologic surgery, as robotic technique improves with each surgery and across different types of surgeries. Beyond concurrent development of robotic surgical technique, our learning curve encompasses the LUAA technique development and maturation, with subsequent improvement in decreasing complication rates. These technical advancements have been previously described by our group.^17^ Primary advancements have targeted the UV junction by placement of a U stitch, placement of an alignment stitch at the apex of the detrusor tunnel and incorporation of the adventitia to prevent ureteral slippage. Incorporation of these elements likely resulted in transient increases of operative time during development. However, overall implementation of these modifications likely improved outcomes of the surgery without significantly disrupting progress in developing surgical skills. In summary, further experience with robotic surgery with the development of the LUAA technique likely concurrently contributed to this learning curve.

This study has a few limitations. The retrospective design introduces the potential for selection and information bias. As a single‐centre, single‐surgeon series, the generalizability of the learning curve parameters to other surgeons and institutions may be limited, as individual learning trajectories are influenced by prior laparoscopic and robotic experience, training environment and case volume. Further, the absence of a concurrent open reimplantation comparator group limits direct efficacy comparison, though the existing meta‐analytic literature provides an appropriate external benchmark.^8^

## 5 CONCLUSION

This study demonstrates that RALUR via the LUAA technique yields high success and low complication rates, with surgical refinement improving trifecta outcomes. CUSUM analysis shows competence is achieved by case 28 and mastery by case 99, with safety maintained throughout all phases. These findings provide a quantitative framework to guide training and credentialling in RALUR. Prospective studies are warranted to validate these learning curve benchmarks across institutions.

## AUTHOR CONTRIBUTIONS

Conceptualization: MSG; Methodology: MSG, PY, DK; Data curation: DF, JA, DK; Statistical analysis: PY; Data analysis and interpretation: DF, PY; Writing—original draft: DF, PY; Writing—review/editing: DF, PY, MSG, DK; Administrative support: MSG, PY, DK; Supervision: MSG.

## CONFLICT OF INTEREST STATEMENT

The authors declare that they have no conflicts of interest.

## Data Availability

The data that support the findings of this study are available on request from the corresponding author. The data are not publicly available due to privacy or ethical restrictions.
